# The Human Vomeronasal (Jacobson’s) Organ: A Short Review of Current Conceptions, With an English Translation of Potiquet’s Original Text

**DOI:** 10.7759/cureus.2643

**Published:** 2018-05-17

**Authors:** George S Stoyanov, Boyko K Matev, Petar Valchanov, Nikolay Sapundzhiev, John R Young

**Affiliations:** 1 Department of General and Clinical Pathology, Forensic Medicine and Deontology, Medical University – Varna "Prof. Dr. Paraskev Stoyanov", Varna, BGR; 2 Student, Faculty of Medicine, Medical University – Varna “prof. Dr. Paraskev Stoyanov”, Varna, BGR; 3 Anatomy and Cell Biology, Faculty of Medicine, Medical University – Varna “Prof. Dr. Paraskev Stoyanov”, Varna, Bulgaria, Varna, BGR; 4 Department of Neurosurgery and Ent, Division of Ent, Faculty of Medicine, Medical University Varna "prof. Dr. Paraskev Stoyanov", Varna, BGR; 5 Consultant Otolaryngologist, North Devon, Uk

**Keywords:** vomeronasal organ, m. potiquet, history, jacobson's organ

## Abstract

The vomeronasal organ (VNO) is a structure located in the anteroinferior portion of the nasal septum and is part of the accessory olfactory system. The VNO, together with its associated structures, has been shown to play a role in the formation of social and sexual behavior in animals, thanks to its pheromone receptor cells and the stimulating effect on the secretion of gonadotropin-releasing hormone. The VNO was first described as a structure by the Dutch botanist and anatomist Frederik Ruysch in 1703 while dissecting a young male cadaver. This finding, however, is widely contradicted due to no elaborate descriptions being made by the Ruysch. The description of the VNO is more widely attributed to the Danish surgeon Ludwig Jacobson, with whom the VNO has been synonymized, as in 1803 he described the structure in a variety of mammals. Whilst Jacobson extensively studied prior reports of the VNO, he publicly denied its existence in humans. Following these discoveries and some contradictory statements in 1891, M. Potiquet published one of the more influential reviews on the topic. To this day, despite the first report of the organ's existence being made in a human and many articles stating its presence and supporting its function, the presence of a VNO in humans is still widely debated upon.

## Introduction and background

In humans, the vomeronasal organ (VNO), also known as (Jacobson’s) organ is an accessory olfactory organ situated on the anteroinferior third of the nasal septum [1]. It consists of a blind sac with a duct opening anteriorly, both supplied with a rich vascular and glandular network. The organ contains specialized olfactory sensory cells or esthesiocytes, which function both as afferent neurons in the reception of pheromones via the terminal cranial nerve and also produces gonadotropin-releasing hormone.

## Review

Discovery

The VNO was first recorded by Dutch anatomist Frederick Ruysh, who made a very clear depiction of it in his line diagram of the dissection of a recently deceased two-year-old male cadaver [1]. The diagram depicts a vomeronasal organ; however, Ruysh never made any specific reference to it.

Over the following decades, other anatomists made mention of this same structure [1,2]. However, since the VNO was much larger in domestic and wild animals, research was primarily aimed at the mammalian counterpart.

The vomeronasal organ was formerly known (and often still is) by the eponymous name of the Danish anatomist, Ludwin Jacobson, later translated into anatomical Latin (and then English), by the Swiss anatomist Wilhelm His as Ludwig Jacobson [3]. Much confusion still surrounds Jacobson’s contribution and over the years he has been misquoted by a number of subsequent researchers [4,5]. This could well be due to the fact that Jacobson’s original seminal work, published in 1813 was not very accessible; it was printed in Gothic script and in Danish and remained almost unknown [6]. It was not until 1950 that it was translated into French by Danish scholars, but only 150 copies of that version were made and it too remained mainly unacknowledged. Eventually, it was discovered in the library of the Agricultural University of Copenhagen and translated in 1998 by two Europeans, Trotier and Døving [7].

The title of Jacobson’s paper was “Anatomical Description of a New Organ in the Nose of Domesticated Animals” and there is no dispute about his discovery of the anatomical feature in mammals [6,7]. Where the later confusion arises is when subsequent workers refer to humans. Jacobson neither discovered the human vomeronasal organ nor did he refute its existence in homo sapiens: he mentions it twice in his paper. He says, “in the monkeys, it becomes so small that we are prepared to see it vanish completely in man” and further, that humans, who possess a very well-developed sense of taste, have only a rudiment of the organ.

Human vomeronasal organ

**Figure 1:**
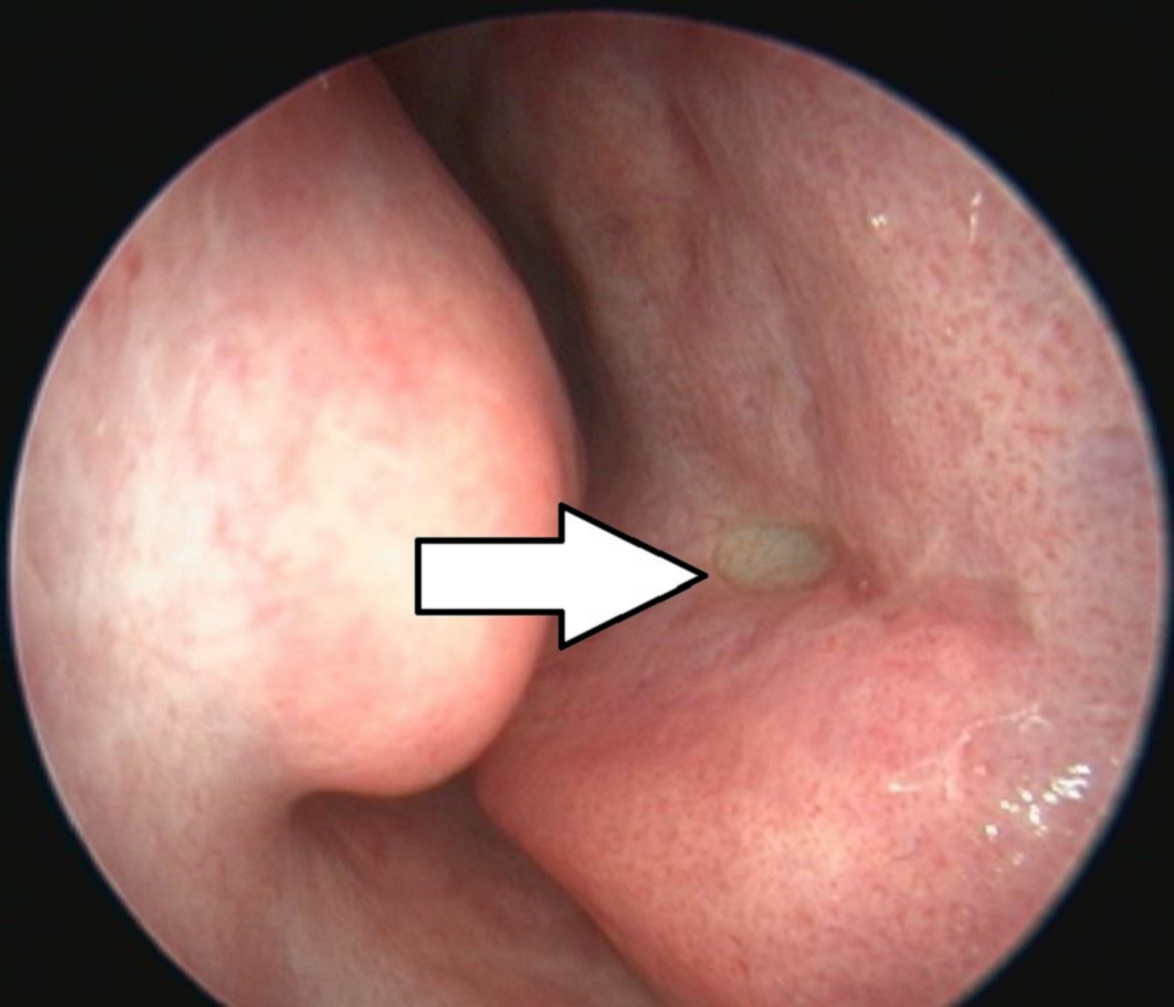
Endoscopic view of the human vomeronasal organ located on the right side of the nasal septum (arrow)

**Figure 2:**
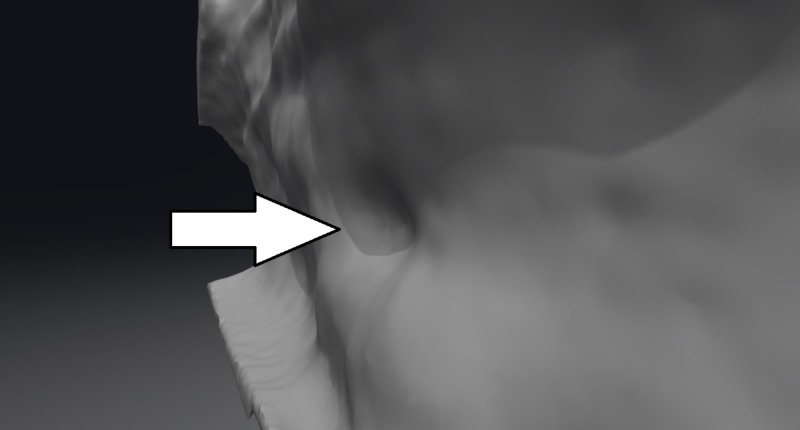
3D reconstruction of the nasal septum from a CT scan of the patient from the previous figure with the vomeronasal organ visible (arrow) 3D: three dimensional; CT: computer tomography

**Figure 3:**
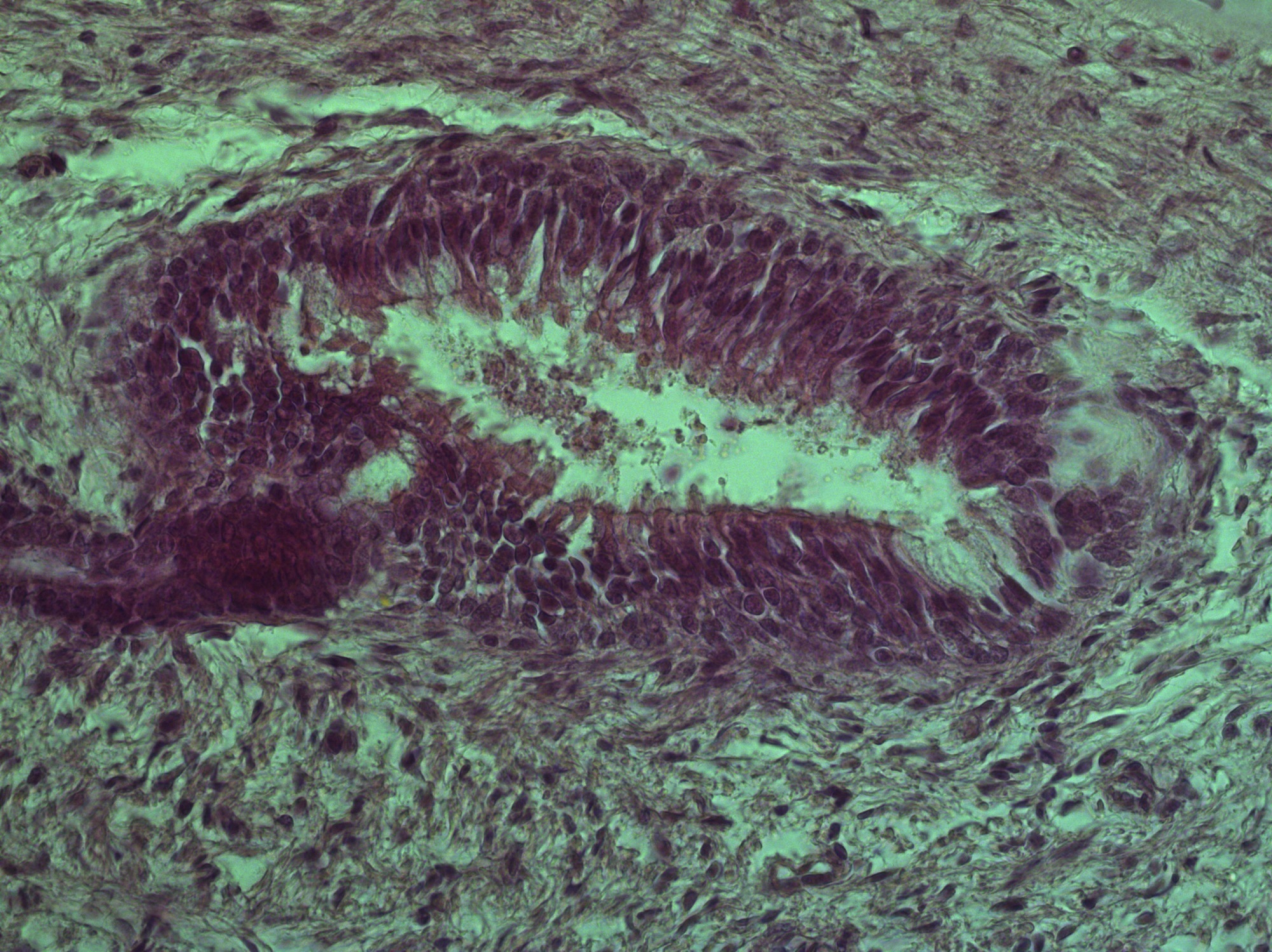
Histology of the human vomeronasal organ on the nasal septum of a 17th gestational week human fetus with ciliated estheziocytes, supporting cells and ganglion cells Hematoxilin and Eosin, original magnification 400x

The function (and in some cases the existence) of Jacobson's organ is still the subject of heated controversy [8]. There is little doubt however that the VNO is present in humans (Figures 1-3). Since its discovery, it has been reported using different modalities: direct and endoscopic observation (in vivo and cadaveric); imaging modalities – computed tomography (CT) and magnetic resonance imaging (MRI) (with and without contrast); histological – classical stains and on electron microscopy [9-15].

Despite this abundant evidence, this small and insignificant anatomical feature is nonetheless often still overlooked by contemporary otorhinolaryngologists in the clinical examination of the anterior nares.

Although a constant incidence has been found in different adult populations around the world (Bulgaria, Canada, Egypt, France, Mexico, and the United States of America), studies have shown it to be present (at least unilaterally and predominantly on the left side) in around one third of the population [9-11, 15-18]. Reports suggest that it is more commonly found in children [6, 16-18]. Some studies claim that it is present in over two-thirds of young people, with an increased incidence bilaterally, whilst other researchers suggest that it is present on both sides of the septum in almost all newborn babies [14, 18-19].

Function in humans

Human studies using an evoked electrical potential in the nasal mucosa (electrovomeronasogram) have claimed to demonstrate a definite receptor function of the VNO, but there is also genetic evidence to the contrary claiming that genes which code for vomeronasal receptor proteins and the specific ionic channels involved in the transduction process are mutated and nonfunctional in humans [19].

Claims that surgery in the septal area of Jacobson’s pit might cause possible changes in sexual behavior are clearly of great concern to rhinologists [19]. These worries are reinforced by the findings that the neural bodies in the terminal nerve liberate gonadotrophin-releasing hormone in response to stimulation of the VNO.

The connections of the terminal nerve (also known as cranial nerve N) to the VNO is the latest factor in the ongoing controversy surrounding Jacobson’s organ [9]. Although the nerve has received much attention in non-primates (where it is relatively much bigger in size), studies of both its structure and function have been comparatively neglected in humans [20-21].

Another recent interesting finding is the implication that the accessory olfactory system may play a role in the development and treatment of post-traumatic stress disorder.

Yet another intriguing suggestion was made in 1891 in this paper by the French surgeon, Potiquet [22].

After more than three centuries of debate on the topic, the vomeronasal organ continues to incite controversy and argument.

Questions undeniably remain unresolved about its function in humans and its role in the development of sexual and social behavior as a whole. However, there are some indisputable facts. One such is the presence of ectopic esthesioneuroblastoma, a rare type of malignant tumor developing from olfactory neuroepithelial cells. This most often arises in the areas containing olfactory neuroepithelium, (adjacent to lamina cribrosa, the superior aspect of the nasal septum, and the superior nasal concha). However, there have been rare reports of this malignancy developing in the area of Jacobson's duct. Since there are no esthesioblasts normally found at this site, this would definitely suggest that the VNO is an accessory nasal area in humans.

Further possible tenuous evidence comes from the theory of the pneumatization of the vomer. It has been posited that a "pumping" mechanism leads to pneumatization of the vomer and in this case, the formation of the vomeronasal duct.

## Conclusions

Although it is a very small and somewhat obscure (and undoubtedly very neglected) anatomical landmark, the vomeronasal organ remains a hot controversial topic for research both to the intimacies of the structure, its functions, and connections to other systems in both humans and animals.

## Figures and Tables

**Figure 4:**
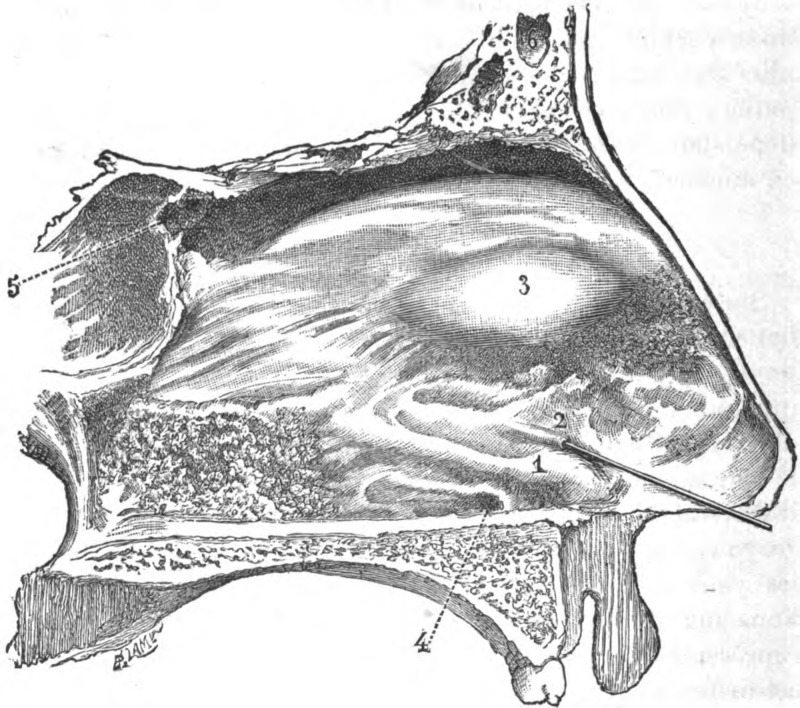
Figure 1 of the original text The septum of the nasal fossae. The septum presented here is not the first one that came. Of the eleven subjects of which we had dissected the nasal fossae last May, we chose this septum for the image, which, quite regular in its form, offered clearly defined and recognizable the anatomical particularities noted here. It belonged to a man of about forty years. The superior lip and a bit of the skin of the under septum were missing in the piece, entrusted to the draftsman. The aforementioned, by our advice, reconstructed schematically these parts, however by sparing the fabric a little too much and the distance of the orifice of the Jacobson's canal from the angle of the under septum and the superior lip is certainly lesser than the real distance l. Bead, constituted in part by Jacobson's cartilage. — 2. The orifice of the Jacobson's canal, in which a stylet has been introduced. — 3. Tubercule of the septum. — 4. Nasopalatine infundibulum responding in the skeleton to the nasopalatine canal. — 5. The orifice of the Sphénoïdal Sinus. — 6. Frontal Sinus.

**Figure 5:**
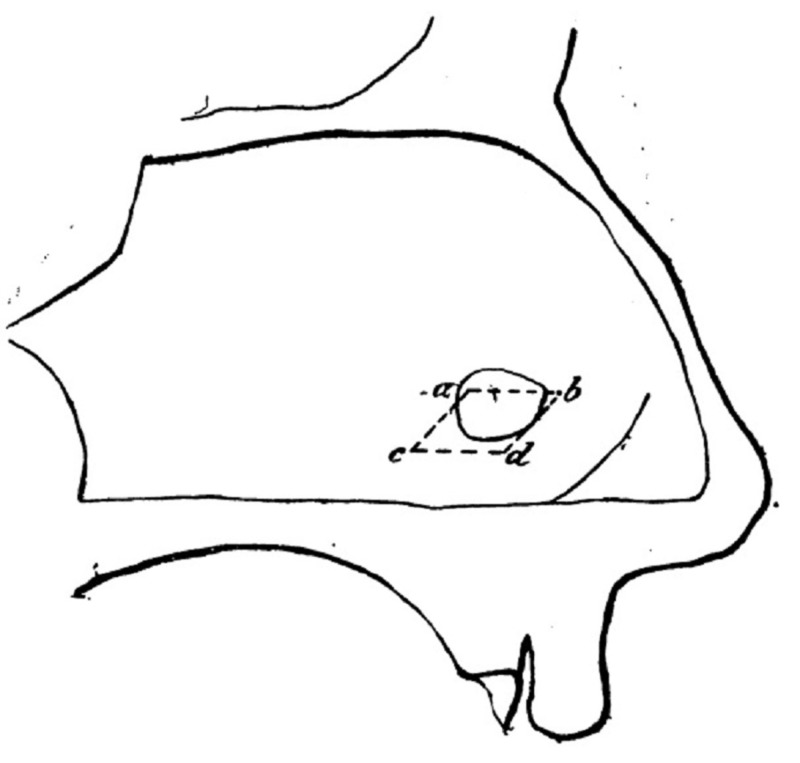
Figure 2 of the original text Graphic of a perforated nasal septum (perforating ulcer, syphilis?) located in a subject of approximately sixty years of age. The dotted rhombus a, b, c, d, on this septum, roughly limits the space in which the orifice of the Jacobson Canal was located in eighteen adult subjects, according to the measurements of M. A. Koelliker

## Appendices

Of Jacobson’s Canal. Of the Possibility of Locating it in Living Beings and of its Possible Role in the Pathogenesis of Certain Nasal Septum Lesions

(Du Canal De Jacobson. De La Possibilité De Le Reconnaitre Sur Le Vivant Et De Son Rôle Probable Dans La Pathogénie De Certaines Lésions De La Cloison Nasale)

By Dr. M. Potiquet

Jacobson's canal in humans represents a vestigial remnant of the organ of the same name. Jacobson's organ, which serves for olfaction, achieves its fullest development in certain mammals. In sheep, for example, it consists of a membrano-mucous tube, enclosing several ramifications of the olfactory nerve; this tube itself is located in a cartilaginous case, applied on each side of the septums of the nasal fossae. In humans, the organ is only rudimentary: the cartilaginous case is reduced to thin strips or small rods of cartilage (Jacobson's cartilages, accessory cartilages of M. Sappey), which follow the base of the quadrigeminal lamina and the apex of the vomer (These cartilages play an important role, indicated by M. Sandmann (International congress of Berlin, 1890) in the width of the antero-inferior portion of the septum, a role which we will try to define in a future study) on each side, and what remains of the membrano-mucous tube is a canal or a slightly extended cul-de-sac, lying underneath these cartilages, therefore located towards the lower part of the cartilaginous septum.

Noticed and described in humans by Fr. Ruysch (Thesaurus anatomicus, tome III, 1703) and later by S. Th. Soemmering, in his magnificent graphics on the anatomy of the olfactory organ (Abbildungen d. menschlich. Organe des Geruches, 1809), this small canal has been, since the discovery of Jacobson’s organ in mammals (Annals of the Museum of Natural History, tome XVII, Rapport by Cuvier) (1811), mentioned in humans by J. Fr. Merkel (Handbuch der menschlich. Anatomie, tome IV, 1820. Cited by A. Koelliker) and studied in the human embryo by Dursy (Zur Entwickelungsgeschichte des Kopfes des Meuschen, etc., 1869). In 1877, M. A. Koelliker makes Jacobson’s organ in humans the subject of a monograph (Ueber die Jacobsonschen Organe des Menschen, etc., 1877), and afterward, one can find this small canal cited or studied in the articles of M. Th. Koelliker (Ueber des Os intermaxillare des Menschen, 1882), Shwalbe (Lehrbuch der Anatomie der Sinnesorgane, 1887), Loewe (Monatsscb. f. Ohrenheilln, 1886, and International Congress of Berlin, 1890), Quain (Monatsscb. f. Ohrenheilln, 1886, et congrès internat. de Berlin, 1890), Zuckerkandl (Real-Encyclopedia der gesammten Heilkunde, 2e édition, 1888. Art. Nasenhôhle), etc. (As M. Loewenberg had remarked in the discussion, following this communication, M. G. Gegenbauer had contested the significance attributed to Merkel, Dursy, A. Koelliker, etc., of this cul-de-sac. He sees the rudiment of a very developed gland in certain prosimians (Morpholog. Jahrbucb, 1885). As far as I know, it has not been described or presented in any French works. At the very least, neither Gratiolet (Researches on Jacobson's organ. Thesis of Paris, 1845) nor M. Ch. Remy (The mucous membrane of the nasal fossae. Dissertation, 1878), describe it in humans.

Its existence in man is constant, claims Soemmering. We would not be as affirming, at least in regards to adults or the elderly. During recent dissections (May 1891) in eleven heads of adults or elderly people of relative freshness, we have found it eighteen times (We are glad to thank here M. Poirer, chief of the anatomical works of the Faculty, who had authorized our research). The search of its orifice is relatively easy in cadavers. The eye, when the orifice has been carefully cleaned, when brought to light, placed in front or inclined in different directions, can freely observe all the inequalities of the mucosa, if needed one can employ a scalpel, and finally discover in the indicated space a rather large crypt, which is nothing else than the sought canal. In the head of a newborn or of a child of several months, it is always found, often preceded by a small furrow leading up to it. To us, it seemed that in adult heads it was more likely to discover it if the subject was younger: this without a doubt is due to lesions of the mucosa, dependent on repeated coryzas, which probably with age lead to its obliteration (However, we have found it quite frequently in the ozaena-afflicted).

In the living, it is slightly more different. M. Moldenhauer is, to our knowledge, the sole rhinologist who has tried to find it and his research was in vain: "Despite my attention being directed to this point many times", he says while speaking about the orifice of Jacobson’s canal -"I could not see it in the living (Maladies of the nasal fossae, works translated by us, 1888, page 43)." He describes it surrounded by a bead, he is correct; however, in adolescents and in adults, if we believe the anatomic pieces that we have had before our eyes and our findings in the living, if we have faith in the figures of the septum in which it is found (Soemmering, A. Koelliker, Schwalbe, Merkel) (Figure borrowed from M. Merkel by Zuckerkandl), this orifice presents itself mostly limited by a valvula; the bead does not seem to exist at all but in the youngest of children (See the figure in Ruysch's work), and if M. Moldenhauer did not manage to locate it in the living, it is probably because he had begun looking for a bead as a landmark.

The orifice of this small canal, which, most often, deserves only the name of a cul-de-sac for its slight elongation, is however not impossible to find in the living. Its discovery here is certainly less easy than in a cadaver, but with some care and on occasion a lot of patience, one can manage to find it, to find it sometimes, we would have said a few months ago, quite often, we say at this hour.

To find it, one must know where to look for it first. It is situated below the bead, stretched from back to front, which comprised mostly by the cartilages of Jacobson, occupies the anteroinferior portion of the septum (Figure 4).

According to the measures of M. A. Koelliker, it is 8 mm long on average, located 5 mm from the roof of the nasal fossa and 23 mm from the angle formed by the membranous septum and the upper lip, it is 1 mm large. The length of the canal directed from the front to back and slightly upwards in which it gives access can reach up to 9 mm (Schwalbe), but on average it measures at around 4 mm.

These are only averages; Jacobson's canal can open a bit closer or a bit further than the aforementioned corresponding values. Following the minimum and maximum distances indicated by M. A. Koelliker, one can define a roughly rhombic shape over the septum (Figure 5), measuring 7 millimeters from base to top, 8 millimeters from back to front, in which we can almost certainly find the orifice. This space can be titled the search area; further anatomic studies will without a doubt enlarge it, the measurements of M. A. Koelliker were conducted only on 18 adult subjects.

In addition, our research on the corpse has taught us that Jacobson's channel is, in a single subject, quite symmetrically placed to the right and to the left, at a distance very substantially equal above the floor of the nasal fossae. However, its length and also the distance, separating its orifice from the nostrils, and its situation from back to front are subject to several variations from one side to the other. This equal distance from the roof of the nasal fossae to the left and to the right in the same subject is a circumstance which could enormously facilitate the discovery of the orifice on one side when it has been found on the other, and also, from a pathological standpoint, it could constitute precise information.

Considering the ease with which one can find the orifice of Jacobson's canal in a cadaver, one could be astonished that its discovery in a living creature could be this difficult; however, in a living subject, the conditions are completely different and exactly they do not fail to be quite unfavorable.

First, for the clear perception of details, no lightning can best direct sunlight. However, under the circumstances, rhinoscopy can only be practiced with the aid of artificial lighting, which changes the color of the objects of interest and blurs their contours.

Second, during anterior rhinoscopy, as commonly practiced, the mucosa of the septum, which is far from being visible up front as it is in cadavers, presents itself obliquely; also, light accidents which move its surface, risk enormously to get lost in a confused shortcut.

Third, the mucosa of the septum is quite often covered, especially at the level of the zone that interests us, sometimes by mucous, spread in a very thin layer, which takes the appearance of varnish due to desiccation, sometimes by heavy quantities of epithelial debris. In that case, this is not the surface of the mucosa which one perceives in reality, but the products of secretion or desquamation, which mask its details.

Fourth, the cartilaginous septum offers quite often, especially in its inferior portion a tormented configuration; even if it looks sufficiently straight, it presents itself often towards the anterior limits of our search zone, or in front of them, a heavy vertical ondulation, which makes its exploration with an eye or a stylet difficult.

These are the principal circumstances that impede the examination of this portion of the septum and which could prevent the discovery of the orifice of Jacobson’s canal.

In regards to the first point, the exploration of the nasal cavities with direct sunlight with a planar reflector can be practiced only by exception.

The second point can be remedied partially by directing the speculum so that our search zone presents itself in view as least obliquely as possible.

A wool tampon affixed at the end of a stylet should be carried over the septum with an utmost lightness to brush off mucous and epithelial accumulation; they should be brushed with infinite softness, as to not provoke blood seepage, which would hamper any further immediate exploration.

The patience of the rhinoscopist, his ingenuity and eventually his experience will aid him in catching, among the multiple accidents of a wavy, mountainous, hilly septum, the sought-after canal; however, very often he will have to resign himself to extracting his stylet empty-handed.

Other than the inherited difficulties of the region, there are some that arise from the canal itself—I would say the narrowness of its orifice and its brevity.

The region that it occupies is one of those which the stylet of the rhinoscopist often traverses; never have I, however, heard of it being engaged. The narrowness of its orifice saves it from any accidental introduction of the commonly employed stylet, too thick to be able to penetrate it. Since this is rhinoscopy, viewing is the obligatory exploration method; but if one employs only it, one risks heavily to deceive oneself. Even in the most favorable cases, the eye can only suspect the orifice of the entry of the canal. A darker spot in the aforementioned place, a slight depression, a furrow breaking off abruptly leads us to think that there lies the sought orifice. But certainty can never be acquired without the stylet. The stylet imposes itself, not at all some random stylet, but a very fine stylet, around six-tenths of a millimeter in thickness, perfectly soft at its extremity. One inserts it lightly in the presumed orifice; if it penetrates the mucosa at 3, 4 millimeters deep or more, one can be certain that one is exploring Jacobson's canal. Its introduction is, moreover, most often absolutely painless. Its extraction could be followed by the appearance of a small droplet of blood at the entrance of the canal if the extremity of the stylet had pressed against the bottom with too much force. However if the stylet glides over the mucosa without penetrating it, one must search elsewhere; and if nothing in the vicinity attracts the view particularly, one must lower oneself to scratch with the extremity of the stylet pressed front to back in the zone that we have traced (Figure 5), even a bit outside of it, especially above the Jacobsonian bead.

If the narrowness of Jacobson’s canal makes the search very laborious, its brevity, which sometimes only reaches 2 millimeters (A. Koelliker), can quite often keep the mind guessing about the true significance of the canal in which the stylet has gone. From a purely morphological point of view, without referring to embryology and comparative anatomy, Jacobson’s canal is above all simply a crypt in the mucosa, a crypt certainly larger and overall deeper in general than those located in the mucosa of this portion of the septum underlying the tuberculum septi. But morphologically speaking, this is just a crypt and when it is superficial, when the stylet, barely engaged, finds itself stuck, a certain doubt comes to mind: has the stylet come into contact with the bottom of an ordinary crypt? Relatively easy to decide on a cadaver, where the eye mainly comes to our aid, where one can freely examine an anatomical piece, use the stylet on it, manipulate it in every direction, the circumstances are not as simple in the living and the examination does not enjoy the same facilitations. However, there is a diagnostic element that the examination of a cadaver teaches us to attach much importance to, which is the seat of the orifice. Jacobson’s canal, despite being deprived of any connection with the cartilage of the same name in humans, has always appeared to us in cadavers and in the undeniable cases in the living, overlaying the bead that of which these cartilages contribute to the formation of, at the base of the septum, or a bit above it—it is there that the septum offers vertically and transversally its most significant thinness. On the other hand, if the distance above the roof of the nasal fossae varies from one subject to another, in its quite restrained limits, it is correct, that this distance is, as we have noted, significantly the same from one side to the other in the same subject: precise information, we said, that everything being equal facilitates enormously the search for the orifice when it has been found on one side.

This is, as one can see, an often-laborious investigation that is sometimes pointless since Jacobson's canal could have been obliterated; an investigation not always met with success, even when the canal seems to have existed. From the month of February to the month of August 1891, our attempts, which we had pursued ruthlessly, in all of the subjects that have offered themselves to our observation, above all inoffensive, have had positive results in about a hindered case from around a bit more than two hundred subjects: therefore, around one time in four or five investigations (We had, the day before, November the 4th, in a patient of the Saint Louis hospital, Bazin pavillion, room 3, bed 57, shown Jacobson's canal to M. Hallopeau, MM. Bataille and Floerschein, one of his interns, to M. Flandre, one of his externs and to M. le Dr. Le Baron who was present during the visit).

- This is a long time to waste, one would say maybe, and for what meager result! - To insert a small stylet from time to time in a several millimeters hole!

-Should rhinoscopy linger on such an insignificant thing? Is the process even worth the candle? What could pathology possibly gain from this miniscule maneuver? Here is a humble cul-de-sac, left alone since no stylet has thought of visiting it yet. Will a new era open up for him, and the explorations that it will maybe be subject to, will they always be without damage to it and even the septum?

-So firstly, one can reply, any enrichment of the technique, applied in the examination of the nasal cavities is not to be disregarded, as minimal is it may be. According to Czermak, Voltolini and M. Duplay, one cannot deny that the progress of the pathology of these cavities is connected to the perfection of their methods of exploration. How would it be irrelevant to be able to recognize on occasion Jacobson's canal? The search for it could not be, and this goes without saying, an obligatory part of every exploration, even methodical, of the nasal fossae, considering that, in the vast majority of cases, its utility is null. However, in our opinion, it is enough that such a utility exists should the rhinologist in the future need it, why should not Pathology benefit from the supplement of information that this new investigation procures? We are convinced that many lesions occupying the region of Jacobson’s canal and Jacobson’s canal itself could currently be recognized, and therefore stopped in time, because until now they have remained unnoticed because of their insignificant size. As far as we are concerned, we confess to not having noticed the small elongated cicatrix, presented by a syphilis patient the observation of which we will share later, a syphilis patient, undergoing our examinations for a while, before our special attention was drawn to this region. In regards to syphilis notably, it is a fact that strikes when reading the observations, even collected with the assistance of experienced rhinoscopists, it is the frequency of finding an evolving perforation of the septum and the extreme rarity with which we note at this point the presence of ulcerative syphilis, the perforation of which is often only a consequence. It seems likely that a more detailed examination of the region will put an end to this kind of antinomy.

It is possible that repeated intrusions, even with care, could damage Jacobson's canal and sometimes lead to its obliteration. But the damage to the individual could never be too significant, we can agree. Like the ilio-coecal appendix that one removes quite voluntarily when one judges it to be sick, Jacobson's canal in humans is simply an unnecessary organ and the organism does not seem to suffer from its absence. Without going as far as claiming that its obliteration is a useful mutilation, one can state without much hesitation that this is a mutilation without consequences.

M. Holdenhauer, after noting the existence of Jacobson’s canal in the thickness of the mucosa of the septum and relating the failure of his research adds: “Jacobson’s organ does not have any importance for Pathology.

A lot of reasons and some collected observations, notably at the Saint-Louis hospital (Our research from a pathological point of view was performed by M. Hallopeau on the recommendation of our venerated master, M. Lailler, was kind enough to give us a most benevolent welcome. We are happy to address him here with our ardent gratitude) incline us to agree with this judgment.

To say that an organ has no pathological importance is a very bold statement. Alas! There is not a single one of our organs that cannot be sick, one would communally think, and in many ways! And among these organs, the ones, like Jacobson's organ, which exist only in a vestigial state, the ileo-caecal appendix, for example, are maybe not those that oppose with most resistance the processes of sickness. One knows, thanks to the remarkable works of our master, M. Talamon, the relative frequency of appendicitis. Would Jacobson's canal, therefore, constitute a difference? 

Instead of believing in some immunity that explains nothing, would it not be better to simply confess that until now its afflictions have stayed probably unnoticed, and this thanks to its furtive situation in a region which one explores relatively little, the small space that it occupies, the difficulty of recognizing it, and the even bigger difficulty of noticing its lesions from the start, while it is still recognizable and has not been compromised in a slightly extended destructive process; finally, in order to say everything, if until now one has not even mentioned its afflictions, would it not be because of the sufficient reason that one ignores it quite generally, even its existence?

Something of note, the region of the septum, by far the most important from a pathological viewpoint, the one in which the morbid processes, whatever their nature, are localized with the most marked predilection, is precisely the one which shelters the Jacobson's canal. There lie the syphilitic ulcers described by M. Michelson, the perforations from the same cause, the perforating ulcer described by MM. Weichselbaum, Voltolini, Hajec, etc., and which is observed relatively frequently; there is the preferred place of origin of repeated epistaxis, there the lupic neoplasms and the perforations that follow appear with a little known frequency in lupus of the face, the nodules of leprosy and likewise the loss of substance that follow their desegregation, the perforations which become apparent after contain infectious diseases, recurrent typhus, typhoid fever.

A quick look at Figure 5, in this regard, would be particularly instructive. What would one report on this perforated septum by an unknown process (perforating ulcer, syphilis), discovered by us in the cadaver of an man of around sixty years of age, the minimal and maximal distances, noted by M. A. Koelliker, from the roof of the nasal fossa to the orifice of Jacobson’s canal on one part, and of the same orifice to the angle, formed by the membranous septum and the upper lip on the other, one would obtain almost the diamond shape, which was traced in dots and which we have previously titled the search zone. The superposition of this zone and of the perforation, neither abiding by any geometric perfection – one would not want for it for a number of reasons which is impertinent to mention, is it not a topic of heightened interest and these most suggestive quasi-concordances (Ignoring the painting we could not take but a rough sketch on the field of the entire piece with the aid of a compass. The examination of the preserved fragment, on which the perforation is visible immediately adjacent to the Jacobsonian bead occupying the base of the septum, is more demonstrative than the graphic)?

Of this particular localization of the processes of illness in a determined region of the septum, there has been no general explanation until now. It is commonly stated, regarding the perforations of the septum that their location is such that they are at the level of the thinnest cartilage. But this explanation touches more or less the ease with which the process engages the entire thickness of the cartilaginous septums; it does not at all state why the initial lesion, of which these perforations originate, affects this region more than any other. Wouldn't Jacobson's canal be for some reason in the undoubtable preference of a number of these lesions for the region that is its? Does it play some role in it? Is it not its presence that attracts and fixes them there?

Considering the habits of some of these lesions, the places where they install themselves with a preference, their pathophysiology even their initial forms on the septum, such a proposition seems believable. Regarding the perforating ulcer, notably its seat and form, as well as the seat of the perforation that it can lead to, and the symmetry of the two sides of the septum, noted in a case by M. Hajek (Virchow's Archive, tome 420, 1880), do they not lead us to think that Jacobson’s canal could not be, in certain cases, related to their appearance?

M. Hajek, on the basis of the colossal number of cocci which inhabit the nasal cavities, even in a normal state, makes from this ulcer a necrotic affection of the mucosa, caused by the penetration in the depth of these glands of staphylococci and streptococci pyogenes. “It is self-explanatory that the large excretory conduits of the mucosal glands are particularly exposed to receiving within them damaging agents leading to a local irritation and inflammatory alterations and the coverage with the epithelium of the mucosal glands and their excretory ways. Also, the inflammatory products that reside in the excretory ways, the blood coagulation which, after a hemorrhage, could be formed there, provide a particularly favorable terrain for the multiplication of bacteria which are found there and for their pathogenic action." If this is the pathogenesis of the perforating ulcer, does the role of host, attributed by M. Hajek to the glands of the mucosa, not fit marvelously Jacobson’s canal, which is not much more in humans than a cul-de-sac veritably without any use (Elsewhere, we see another microorganism, the gonococcus, having a natural tendency to take refuge in the crypts of the urethra, to multiply there to then bring a whole series of modifications leading to a phlegmasic process, folliculitis. The favorite seat of this folliculitis would be, according to M. Lefort, Paris thesis, 1888-1889, the Guerin's valve located about a centimeter and a half from the meatus. If the pathogenesis of the perforating ulcer is that of Mr. Hajek, could the cocci, so numerous in the nose, not be able, under certain circumstances, to use the Jacobson's canal to cause a folliculitis, or more appropriately, a jacobsonitis, sometimes resulting, due to the anatomical conditions of the region, to the necrosis of the underlying cartilage? In any case, the analogy is piquant and the approximation was necessary)?

One could be amazed that M. Hajek has not, in his remarkable study, attributed even the smallest of roles to Jacobson’s canal, nor has he thought of it, did he do so to deny any importance and erase it. Does one not know however that the clinic is sometimes a bit slow to utilize the new notions provided by the anatomists? Does the history of the ulcer, perforating the septum, not provide an example of this? Despite being described since 1882 with remarkable clarity by an anatomic-pathologist, M. Weichselbaum and by M. Zuckerkandl, the perforating ulcer continued to be unknown clinically until the moment when Voltolini (1888), Rossbach (1889) and Hajek (1890), created a distinct nosological space of it.

This is not to say, however, that the lesions of the inferior portion of the cartilaginous septum must invariably start from the cul-de-sac in question. It simply provides particularly favorable conditions for their birth, due to its configuration and maybe its quality as a rejected organ.

The process could, without a doubt, be fixed elsewhere (July 8th, 1891. M. R ..., twenty-six-years-old, a copper turner, syphilitic for three years and tuberculous, noticed eight days ago that he had the septum of his nose perforated. Since the month of January 1891, he had at the entrance of the nasal fossae adherent crusts which annoyed him, which he tore out and whose detachment brought light nosebleeds. Perforation of the partition of the diameter of a lentil, occupying the Antero-inner portion of the cartilaginous septum, healed, except in its upper portion, with thinned edges; the mucosa covering them has a cicatricial appearance extending a little beyond. On the left, the Jacobson canal, about 5 millimeters deep, about 4 millimeters behind the posterior edge of the perforation).

But these are only inductions and likelihoods, and it is not with such fragile materials that pathology could be built. We have the sharp suspicion that Jacobson’s organ does not at all escape the common rules, that it could, like all of our organs, even the rejected ones, be touched primitively by certain processes of morbidity, and that its role in the pathogenesis of certain perforations of the cartilaginous septum notably, is far from insignificant, but this is but a suspicion. We have seen lupic and syphilitic cramped lesions, limited exactly to the region that Jacobson’s canal usually occupies, and even, in two cases (1st case. - Bazin Pavilion, Room 2, No. 43, June 1891. A man with multiple tertiary syphilitic accidents; tertiary syphiloma of the superior pharynx; elimination of a part of the sphenoid wings, perforation of the palatal vault, etc. In the left nasal fossa, there is a small scar of the mucosa, greyish-white in color, about 4 millimeters long by 2 millimeters high, occupying exactly the Jacobson's duct above the jacobsonian rim. 2nd case - Bazin Pavilion, 2, No. 95. Mr. X ..., twenty-three years, April 1891. Lupus vulgaris of the lobe of the nose and upper lip, extending to the right in the vestibule of the nasal fossae. In addition, to the right and left, in the nasal fossa, red granulations, bleeding, occupying the area of the Jacobson Canal; the stylet, strolling through them towards their center, crosses the septum from one side to the other. Repeated cauterizations with silver nitrate in substance. In June, granulations disappeared and perforation of the septum with scarred margins, perforation elongated horizontally above the jacobsonian bead, being 4 to 5 millimeters long and about 2 millimeters high, occupying exactly the region where Jacobson canal usually lies), reminding of its form in a shocking way; but we could not monitor them from their very beginning, this is to say that the trajectory of the canal was still recognizable; and the last condition it seems to us that must be met, before one could affirm the existence of a new nosological genre, Jacobson’sitis.

The statement above can be summarized as follows:

1. Jacobson's canal can, if not always, at least quite often, be recognized on the living.

2. Its research has now its place marked in the exploration of the nasal cavities.

3. It is necessary to look for the relation that can exist between Jacobson’s canal and the lesions occupying the area where it sits.

Translator’s comments:

No attempt was made for a literary translation, with the goal to maintain original Potiquet’s ideas, as expressed in French. Throughout the text, there were numerous footnotes; in the translation, all of the footnotes have been provided in brackets, as most of them are akin to comments, rather than references and sources of confirmation.
